# An MRI-Compatible System for Characterizing Supraspinal Processing of Walking-Related Foot-Sole Somatosensory Stimulation

**DOI:** 10.1109/TNSRE.2025.3555852

**Published:** 2025-04-16

**Authors:** Hao Yue, Bin Shen, Yishu Chen, Yufeng Zhang, Jiaojiao Lü, Shaobo Li, Brad Manor, Weijie Fu, Junhong Zhou

**Affiliations:** School of Mechanical Engineering and the State Key Laboratory of Public Big Data, Guizhou University, Guiyang, Guizhou 550025, China; Key Laboratory of Exercise and Health Sciences of Ministry of Education, Shanghai University of Sport, Shanghai 200438, China; School of Psychology, Shanghai University of Sport, Shanghai 200438, China; Key Laboratory of Exercise and Health Sciences of Ministry of Education, Shanghai University of Sport, Shanghai 200438, China; Key Laboratory of Exercise and Health Sciences of Ministry of Education, Shanghai University of Sport, Shanghai 200438, China; School of Mechanical Engineering and the State Key Laboratory of Public Big Data, Guizhou University, Guiyang, Guizhou 550025, China; Hebrew SeniorLife, Hinda and Arthur Marcus Institute for Aging Research, Harvard Medical School, Boston, MA 02131 USA; Key Laboratory of Exercise and Health Sciences of Ministry of Education, Shanghai University of Sport, Shanghai 200438, China; Hebrew SeniorLife, Hinda and Arthur Marcus Institute for Aging Research, Harvard Medical School, Boston, MA 02131 USA

**Keywords:** Sensorimotor control of walking, MRI-compatible foot-sole stimulation system, supraspinal activation, block-designed functional-MRI

## Abstract

Foot soles are the only part in direct contact with the ground during walking. The mechanoreceptors on foot soles continuously obtain somatosensory information (e.g., ground reaction forces) that is delivered to spinal and supraspinal networks. The timely and accurate supraspinal processing of such information, which can be captured by the activation of the supraspinal regions, is critical to the regulation of walking. However, little is known about supraspinal somatosensory processing related to walking. Characterizing the supraspinal response to walking-related somatosensory inputs using MRI is challenging, because individuals are required to stay motionless during MRI scan. We thus developed a stimulation system that simulates the amplitude and timing of foot-sole pressure changes experienced during each step of overground walking, without inducing significant head motion. In the study to examine its validity and reliability of simulation, seven younger adults completed two trials of eight-meter walking. The temporal changes of foot-sole pressure of each step during walking were recorded using a pressure insole and used to program the motion of the system. The results indicated high validity and reliability of the stimulation (rho=0.94∼0.98, p*<*0.0001). Phantom imaging test revealed that the signal-to-noise ratio of the MR image when the system working was similar to when the system was off, suggesting excellent MRI compatibility. Finally, block-designed test indicated that, compared to rest, multiple supraspinal regions (e.g., postcentral gyrus) were activated (p*<*0.005) by foot-sole stimulation. This MRI-compatible system provides a novel approach to characterizing the supraspinal sensorimotor control of walking via MRI.

## I. INTRODUCTION

Sensorimotor control of the lower extremities is critical for maintaining balance during human walking [1]. The foot soles are the only point that is physically in direct contact with the environment while walking over the ground. Mechanoreceptors in the foot sole continuously respond to the ground reaction forces during the stance phase of each gait cycle. These afferent signals are involved in sub-cortical reflex loops [2], and are also delivered to supraspinal networks including the cortical networks within the brain, where they are integrated with other sensory inputs and utilized to form and carry out appropriate motion command [3], [4], [5], [6]. Therefore, the supraspinal response to walking-related foot-sole somatosensory input is likely critical to locomotor control [7], [8]. However, very little is known about the supraspinal processing of these inputs and how it may change with age, injury, or disease.

Several neuroimaging tools have been developed to explore the supraspinal control of human walking [9], [10], [11]. Functional magnetic resonance imaging (fMRI), for example, non-invasively measures neural activity of the brain by quantifying the intensity of blood oxygenation level-dependent (BOLD) signal during rest condition or in response to a stimulus (i.e., activation) [6], [7]. However, it is challenging to measure the supraspinal response pertaining to locomotor control because the individual’s head must remain motionless throughout the scan. Therefore, several types of MRI-compatible mechatronic devices have been developed to apply a programmed sensorimotor stimulus to the foot soles and/or legs with the goal of mimicking certain aspects of walking without inducing motion of the head [8], [14], [15], [16], [17], [18]. Zhang et al., for example, created and validated a pneumatic tactile stimulator capable of applying programmable, two-point pressure stimuli to small areas on the plantar surface of the heel and toes of one foot. This device and others have proven to not interfere with the MR image quality and reliably and effectively activate several supraspinal cortical regions (e.g., primary sensorimotor cortex) with known involvement in sensorimotor control [17]. However, devices tested in previous studies were only able to stimulate small areas of the foot sole, and/or could only stimulate one leg with limited spatial and temporal resolution. They were therefore limited in their capacity to accurately simulate the spatial and temporal characteristics of the foot-sole pressure waveforms that are experienced by both foot soles during overground walking [17].

We created a novel MRI-compatible, bilateral stimulation system capable of applying plantar pressure waveforms with characteristics programmed for each person to simulate the pressures they experience during overground walking, yet while they lay motionless in the scanner. In this work, we completed a series of tests to (1) assess the similarity between real and simulated foot-sole pressure force waveforms; (2) determine MRI compatibility of the system by examining if the working system would influence image quality; and 3) examine the supraspinal activation pattern as induced by this system in a pilot study consisting of healthy younger adults.

## II. Methods

### A. Design of Foot-Sole Stimulation System

**Fig. 1.**
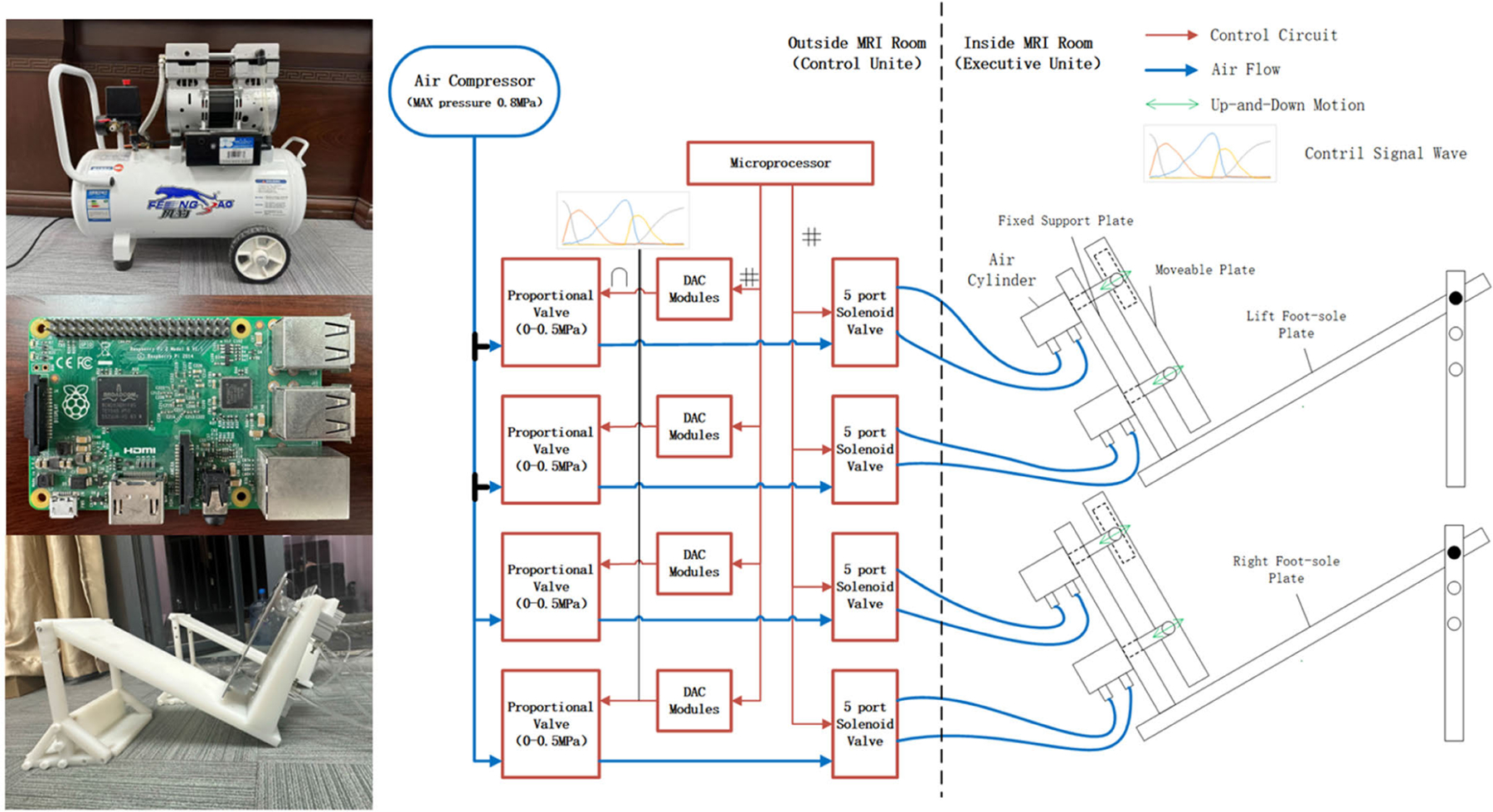
Design of foot-sole stimulation system.

This system consists of the power unit, execution unit, and control unit (Fig. 1). An oil-free air-compressor (Model: OLF750AF, Fengbao, China) that can provide pressure up to 0.5 MPa is used as the source of pressure. The execution unit consisted of two pairs of air cylinders (SDA40×40B, AirTAC, China) (i.e., four cylinders in total), two rigid aluminum movable plates (i.e., one for each side), and one non-ferromagnetic (i.e., nylon) support platform that can adjust the length according to the height of individual and his/her comfort. Each pair of air cylinders is installed on the fixed support plate and attached to a translatable and rotatable joint inside the movable plate. The cylinders can then actuate the plate independently and asynchronously following the command from the control unit, enabling the simulation of the pressure change of each foot sole (Fig. 1). A rigid aluminum rod was attached to a rotatable joint inside the plate through the fixed support plate. During stimulation, the support platform is secured to the scanner table, and the movable plate directly presses against each foot sole with the motion of one degree of freedom to limit the translation of applied pressures to the body and head.

The control unit consisted of a microcontroller (4B, Raspberry Pi, China), a WiFi module, four digital-to-analog (D/A) conversion modules (PCF8591, China), four proportional valves (ITV2030–212s, SMC, Tokyo, Japan), four five-port solenoid valves (4V210–08, AirTAC, Taiwan), and a custom-developed user dashboard (developed in Node-RED). The proportional valves are linked to the air compressor via plastic air tube. The pressure of output airflow is controlled by the direct voltage (VDC) produced by D/A converter as controlled by the microcontroller. The relationship between the pressure of output airflow of each proportional valve and its VDC input is linear, enabling precise control of the magnitude of applied pressure for each cylinder. Each proportional valve delivers the output airflow to the solenoid valves through a tee joint. These solenoid valves shape the airflow wave following the control signal sequences. For example, when the solenoid valve is on, the air input is delivered to the cylinder via its lower port; and when the rapid exhaust is required, the solenoid valve is turned off immediately following the control command. The solenoid valves thus drive the air cylinders to apply programmed oscillations of one degree of freedom. The pressure wave signals are pre-programed based on the pressure time series of each foot sole, recorded by pressure insoles inserted into the shoes during overground walking and processed via the GPIOs (General-Purpose Input/Output) of the microcontroller. The customized user interface enables the design of stimulation parameters (e.g., stimulation frequency and length, the input pressure force magnitude of each valve) and can communicate with the microcontroller remotely via the WiFi modular. The microcontroller can then control the magnitude, frequency, and sequence of pressure applied to each foot sole following the characteristics of the pressure force that is obtained from the real walking. According to the configuration, the cylinder can reverse its movement direction within 2 milliseconds, enabling a maximal oscillatory frequency of 50 Hz. The output force of each cylinder can be up to 0.5MPa.

During the MRI scan, the air compressor and control unit are placed outside the scanner room and concatenated with the air cylinders of the execution unit inside the scanner room via eight, six mm-diameter high-pressure polyurethane tubes. To stabilize the ankle and knee joints during the stimulation and ensure participant comfort, we used a customized medical ankle joint support brace on the support platform with the help of an air bag at the ankle joint. The angle of knee and hip joint is adjustable based upon the comfort of each participant. The ankle joint was fixed around 90 degrees of dorsiflexion to minimize head movements during stimulation following previous studies [17], [18]. Minor adjustment of the dorsiflexion degree may be allowed to ensure the comfort of each participant.

## III. Study Protocol

### A. Simulation of Walking-Related Pressure Changes on Foot Soles Using the Foot-Sole Stimulator

We first examined the capacity of the stimulation system to simulate the pressure to the foot soles that mimic those experienced when walking overground and the step time in a group of healthy younger adults.

#### Participants:

Seven younger adults (age (mean ± standard deviation, SD): 24.1 ± 1.7 years; four women and three men; body mass index (BMI): 22.7±3.1) were recruited and provided written informed consent as approved by the IRB of Shanghai University of Sport. Inclusion criteria included age≤40 years and no history of injuries or falls in the past three months. Exclusion criteria included any acute illness, self-reported history of cardiovascular, metabolic, or neurological disease, musculoskeletal disorders, abnormal shape of foot or history of surgery within the past three months.

#### Study protocol:

Each participant first completed one eight-meter walking test at their preferred speed in a straight hallway. Before the walking test, we inserted a pair of instrumented foot pressure insoles (pedar^®^, Novel, Munich, Germany, www.novel.de) into the shoes to measure the pressure on the foot soles during walking. Each side of the insoles consisted of 99 distributed sensors across the entire plantar surface, and the size of the sensor ranged from 151 to 161 mm^2^. The distributed sensors of pressure insoles recorded the changes of force on different regions of foot soles (e.g., heel and forefoot) during overground walking and the pressure (i.e., the force per unit area) data was then calculated by dividing force by the area of each region via the software of the insole. The sampling frequency of the pressure insoles was 100 Hz. After obtaining the overground pressure data, we programed the pressure applied by the stimulator for each participant using his/her own continuous pressure change data. We used dual adhesive tape to fix the pressure insoles on the movable plate surface of the device to record the force applied on foot soles by the stimulator. Participants were lying supine (similar to their posture in the MRI scanner) and were told to relax their lower limbs. They then completed three stimulation trials. Their foot soles were stimulated at least five times in each trial. Zhang et al. showed that the unilateral pressure intensity of up to 40% of body weight did not induce significant influence on image quality of MRI [17]. Given that this device induces bilateral stimulation, we set the pressure level to 30% of body weight to best mimic walking while minimizing head motion that may interfere with MRI quality.

The temporal changes of pressure on foot soles as applied by the stimulator were then recorded by the insoles in the same way as the real walking test, and the data of such pressure changes from real walking and stimulation were used in the following analysis.

### B. MRI Compatibility Assessment

To examine the influence of the foot-sole stimulation system on fMRI quality, we measured the signal-to-noise ratio (SNR) and signal-to-fluctuation noise ratio (SFNR) following the established fMRI Quality Assurance protocol [19]. We imaged a water phantom in each of the following conditions: (1) ON: the stimulator was powered on and active, implementing randomly-selected pressure stimulation with the intensity of the corresponding body weight, and was placed 80 cm away from the phantom center in the MR scanner room; and (2) OFF: the stimulator was placed the same distance (80cm) away from the phantom center but was powered off and not active.

#### MRI scan:

MRIs were acquired on a 3.0 Tesla Siemens MAGNETOM Prisma whole-body MRI scanner equipped with a 64-channel head coil for radio frequency (RF) reception (Siemens, Munich, Germany). A 3D MPRAGE (magnetization-prepared rapid acquisition gradient echoes) pulse sequence (*T R* = 2300 *ms*; *T E* = 2.26 *ms*; *flipangle* = 8º; *FOV* = 256 × 256 *mm*^2^; *voxelsize* = 1 × 1 × 1 *mm*^3^; 240 contiguous slices) was used to obtain the structural images of the phantom.

#### Data analysis:

All images in each condition were processed using SPM12 (Institute of Cognitive Neurology, London, UK. http://www.fil.ion.ucl.ac.uk). In addition to a visual inspection of artifacts, we quantified the image quality using SNR and SFNR as established in previous work [19]. A 21 × 21 voxel region-of-interest (ROI) placed in the center of the image was created. The magnetic field maps were estimated based on echo-planar imaging (EPI), and the local resonance frequency shift of each voxel was calculated to check the field non-uniformities and the subtle magnetic field perturbations potentially arising from the presence of the stimulator [18], [20], [21].

### C. Assessment of Brain Activation

#### Participants:

Three healthy young participants (two women, one man; age: 23.7±1.2 years; BMI: 22.7±3.6) were recruited and completed this experiment. All of them were screened following the inclusion/exclusion criteria of simulation test and were without contradictions to MRI scan (e.g., medal implant in the brain).

#### fMRI scan protocol:

All participants were instructed to relax their lower limbs. A 3.5-minute block-design stimulation protocol was completed, consisting of three alternating 30-second blocks of rest (i.e., no stimulation) and stimulation applied to both foot soles in alternating fashion as in overground walking. During stimulation blocks, the stimulator applied programmed pressures based upon each participant’s pressure data obtained during overground walking. The amplitude of applied pressure was set at the level of 30% of overground pressures. The blood oxygen level-dependent (BOLD) signal was acquired using a gradient-echo EPI sequence (*T R* = 2000 *ms*; *T E* = 30 *ms*; *FOV* = 210 × 210 *mm*^2^; *flipangle* = 80º; *voxelsize* = 3 × 3 × 3 *mm*^3^; 62 contiguous oblique axial slices, parallel to the AC-PC line; simultaneous multislice acquisition; three runs function scanning). High-resolution structural images were acquired using a 3D MPRAGE (magnetization-prepared rapid acquisition gradient echoes) pulse sequence (*T R* = 2300 *ms*; *T E* = 2.26 *ms*; *flipangle* = 8^◦^; *FOV* = 256 × 256*mm*^2^; *voxelsize* = 1 × 1 × 1 *mm*^3^; 240 contiguous slices).

#### Data analysis:

Images were preprocessed using SPM12 software package (Institute of Cognitive Neurology, London, UK. http://www.fil.ion.ucl.ac.uk) in MATLAB (Mathworks Inc., Natick, MA, USA). To correct for potential head movement between scans, images were realigned with the first scan image and compensated delays associated with acquisition time differences via time correction. Six-parameter head motion curves were obtained. A 2 × 2 × 2*mm*^3^ Montreal Neurological Institute (MNI) template was applied to normalize all images. The activation patterns from the BOLD signal were detected by general linear modeling. The full width/half maximum parameter was set to 8 mm, and the cutoff of the temporal filter was set at 128 seconds. The functional images were spatially smoothed using a Gaussian filter. The head motion was significantly higher in the Z-axis (i.e., the direction of applied pressure) compared to the other directions, but the maximum range of head motion was still less than 1 mm.

### D. Ethics

All the participants provided written informed consent form as approved by the local IRB committee (Pro00044674) in order to participate in all the studies in this work. The potential risk and benefits have been provided clearly to the participants and study personnel confirmed they all understand the study protocol before the enrollment.

### E. Statistical Analysis

All statistical analyses were performed using JMP 16 (SAS Institute, Cary NC).

In each trial of the simulation study, we obtained the actual *entire*force curves of the heel and forefoot of each foot sole as recorded by pressure insoles throughout overground walking and that during the stimulation as applied by the stimulator. The heel and forefoot were the two primary regions of foot soles that perceived pressure during walking. The changes of pressure on these two regions can reflect the transition of pressure on foot sole in a gait cycle (i.e., heel on (when highest pressure on heel, but 0 pressure on forefoot), stance (when the pressure can be observed on whole foot sole, which can be thought as the pressure on heel transits partly from heel to other parts of foot sole, heel off (when 0 pressure on heel, and highest pressure on forefoot), and toe-off (when 0 pressure on the entire foot sole, entering into swing phase). To determine the correlation (i.e., similarity) between the force curves of overground walking and simulation, we used Spearman’s correlation analysis to compare the *entire*curves for each participant. Significant level was set at *p <*0.007 after using Bonferroni correction (n=7) accounting for multiple comparison.

To examine the correlation of step time between overground and simulated walking, the point of maximum pressure applied to the heel of each consecutive simulated ‘step’ was extracted. Then the time interval between these times of highest heel pressure of two feet was calculated and used as the step time [22]. We then used Spearman’s correlation analysis across all the participants to examine the correlation of step time between simulated and overground walking by using each of the step time. We also calculated the magnitude of error of simulation, that is, the difference of step time between simulated overground walking, and used a Bland-Altman plot to visualize this error as a function of the step time magnitude (i.e., the average of the step time in overground and simulated walking) [23]. The significant level was set at *p <* 0.05.

To examine the influence of the stimulation system on imaging quality, we performed one-way analysis of variance (ANOVA) if the data were normally distributed (as assessed using Shapiro-Wilk test) and with homogeneity of variances (as assessed using Levene’s test). If the data were not normally distributed and/or without homogeneity of variances, we used non-parametric Kruskal-Wallis test. The model factor was testing condition (i.e., ON, OFF), and the dependent variables were the image quality parameters (i.e., SNR and SFNR). The significant level was set at *p <* 0.05.

To explore the activation of supraspinal regions in response to the stimulation applied by the stimulator, a fixed-effect analysis was completed by comparing testing and resting blocks of fMRI. The clusters were parcellated using the AAL atlas [8] and Brodmann templates via MRIcron (http://www.MRicro.com/MRicron). Voxel-based significant activation was set at *p <* 0.005 (uncorrected) and the cluster-based significance was set at *p <* 0.05.

## IV. Results

All participants in each study successfully completed all assessments and no adverse events or safety issues related to the use of the foot-sole stimulation system were reported.

### A. The Simulated Pressure Is Closely Associated With That During Overground Walking

**Fig. 2.**
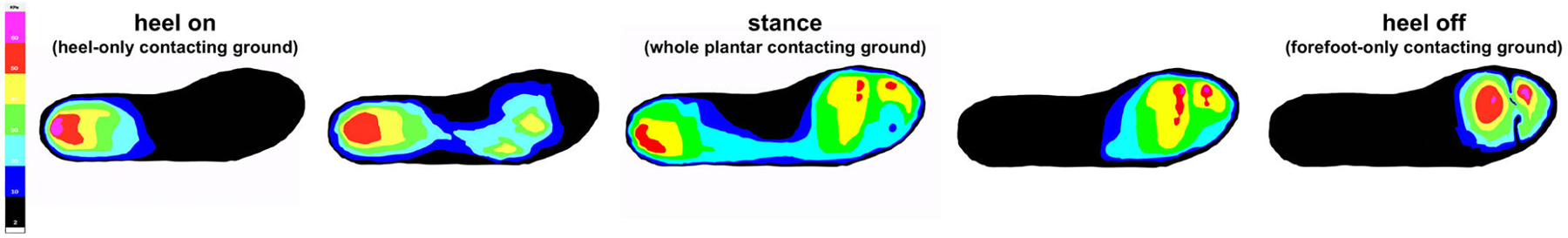
An example of the change of pressure distribution from heel on to stance phase and then to heel off during one gait cycle as simulator by the foot-sole stimulator. The pressure level was set at 30% of the body weight. Warmer color reflected greater pressure.

**Fig. 3.**
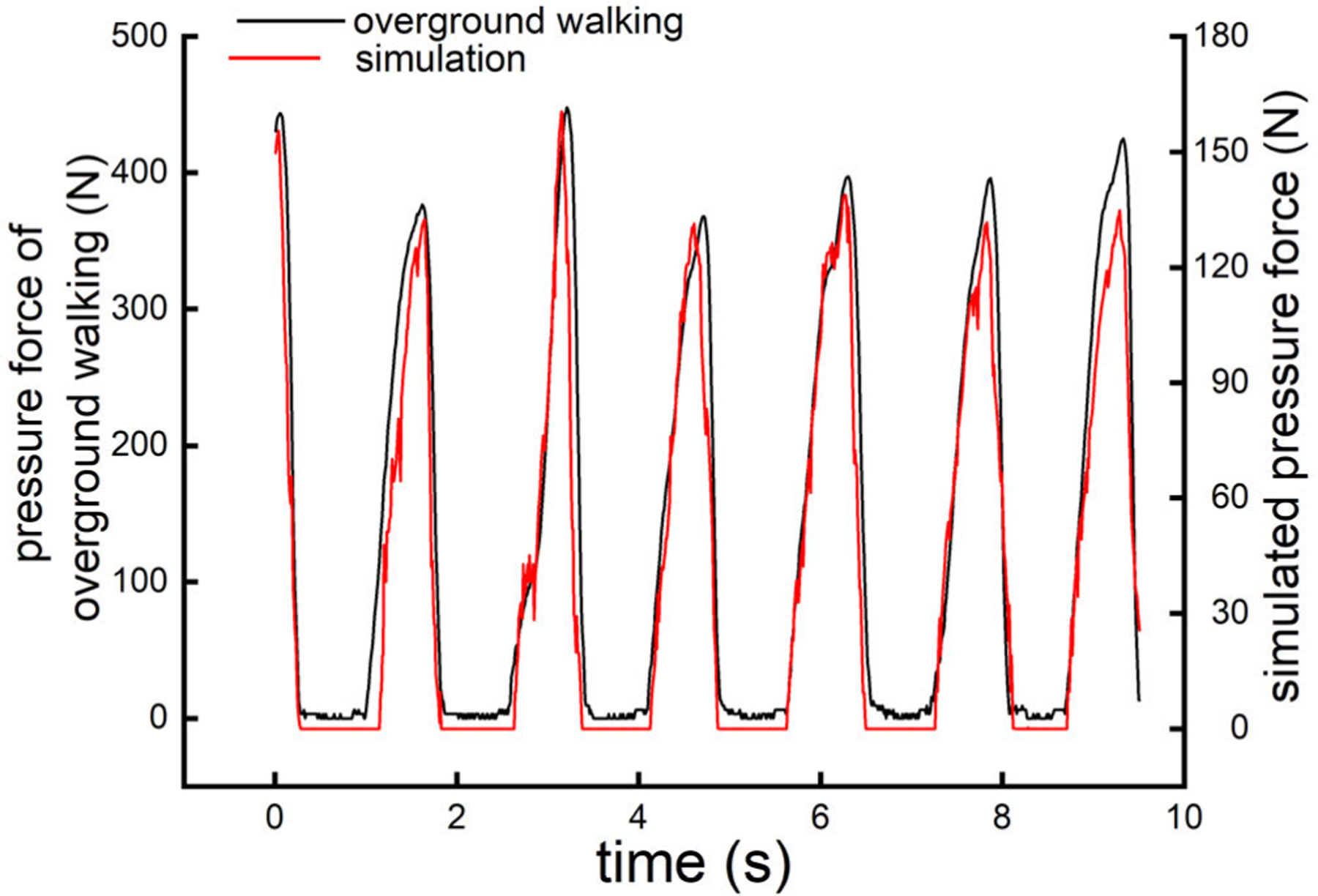
An example of the force changes over the forefoot sole of one foot from the overground walking (in black, axis on the left) and that simulated by the foot-sole stimulation system (in red, axis on the right). The simulated force applied by the foot-sole stimulation system was set at 30% of the body weight.

Fig. 2 shows an example of changes in pressure distribution on one foot sole from heel-on to stance phase and then to heel-off, as simulated by the stimulator. Fig. 3 provides an example of the *entire* pressure curve of the sole of forefoot in one leg from one participant during overground walking and the simulated curve as applied by the stimulator. This suggested that the stimulation system was able to simulate force change as that experienced during the overground walking. Spearman’s correlation analyses showed that the stimulated pressure waves to both the heel (left: *rho* = 0.94 ∼ 0.98, *p <* 0.0001; right: *rho* = 0.95 ∼ 0.97, *p <* 0.0001), and forefoot (left: *rho* = 0.95 ∼ 0.98, *p <* 0.0001; right: *rho* = 0.94 ∼ 0.98, *p <* 0.0001) were significantly correlated with those experienced during overground walking for each participant (TABLE I).

**Fig. 4.**
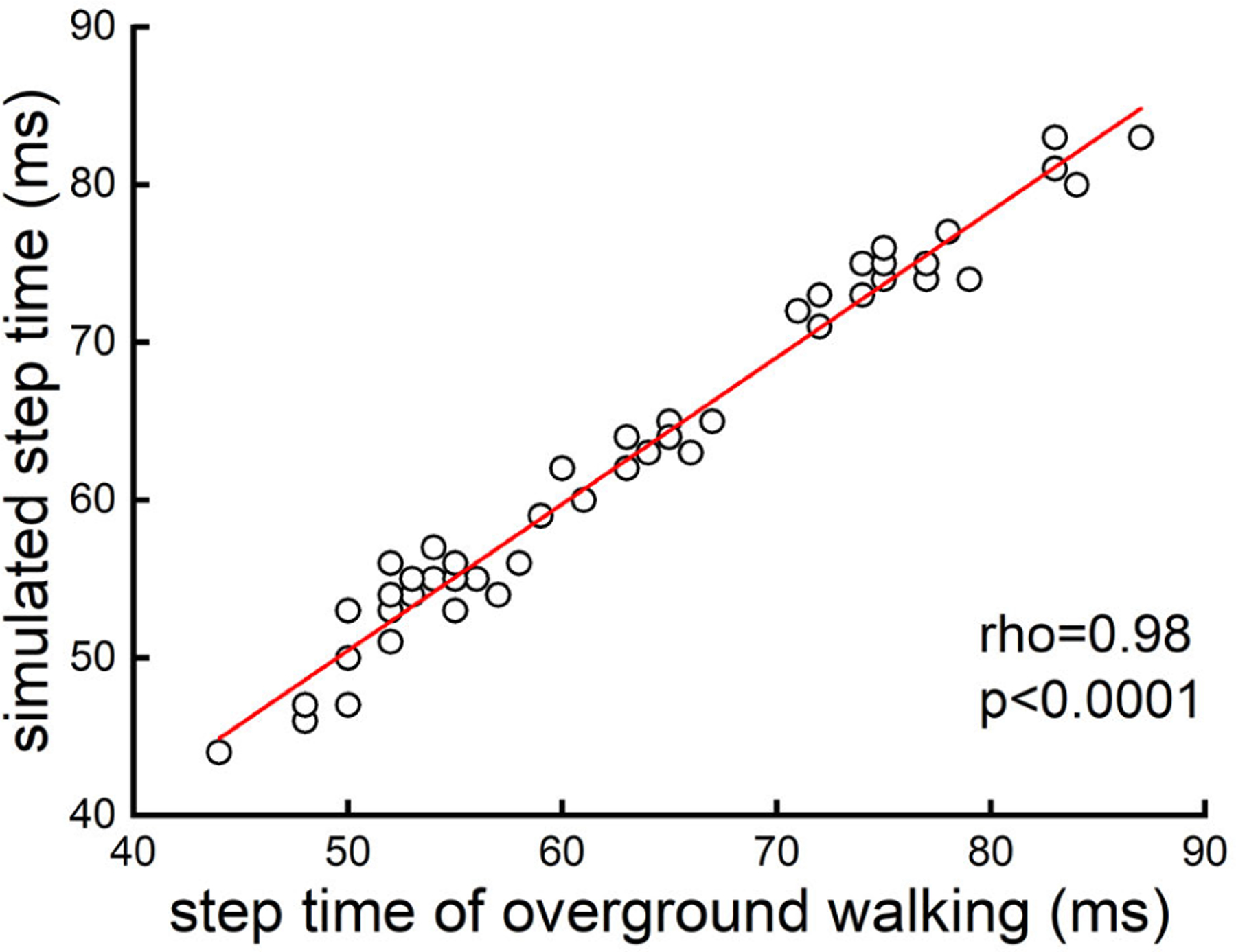
The correlation of step time between simulated and overground walking. A total of 42 steps (i.e., each spot on the figure corresponding to each step) were recorded across all the participants. The Spearman’s correlation analysis demonstrated that the stimulated step time was significantly associated with that of overground walking (*rho* = 0.98, *p <* 0.0001), suggesting that this system can accurately re-produce the step time when walking over the ground.

**Fig. 5.**
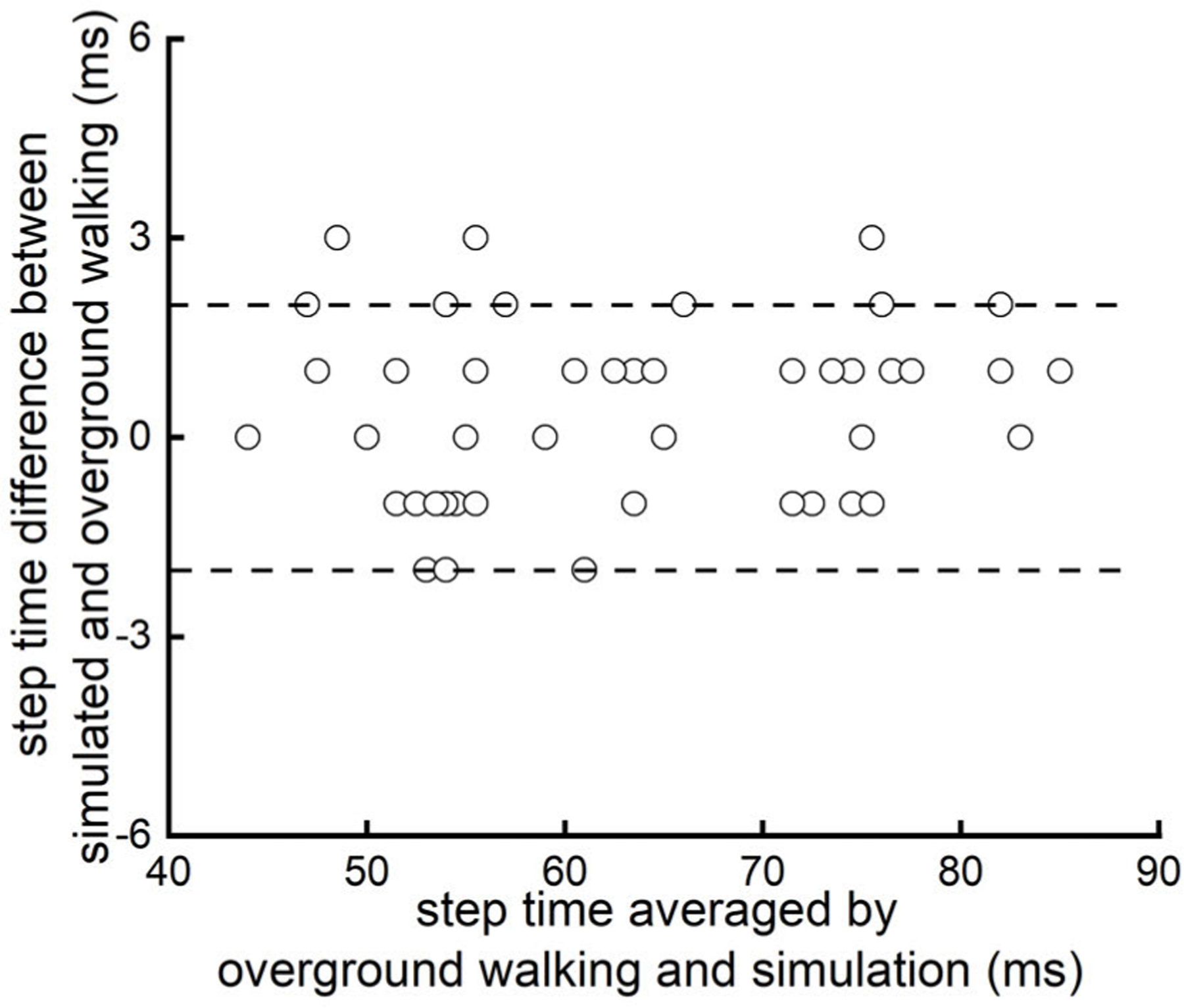
Bland-Altman scatterplot depicting the difference (error) in step time, as a function of the average step time between the simulation and overground walking. The magnitude of error of simulation was not influenced by the value of the step time.

During overground walking, each participant completed at least six steps (six participants had seven steps and one had six steps). TABLE II showed the simulated step time and that of overground walking in each participant. The Spearman’s correlation analyses showed that the simulated step time was tightly correlated with that of overground walking (*rho* = 0.98, *p <* 0.0001) (Fig. 4). The difference between the stimulated step time and that of overground walking was 0.5 ± 1.2 *ms*. The Bland-Altman plot (Fig. 5) showed that the magnitude of error of the simulation of step time was not influenced by the value of step time.

### B. MRI Compatibility of the Foot-Sole Pressure Simulator

The foot sole stimulator had no significant influences on the quality of MR images of phantom (TABLE III). Visual inspection revealed no observable differences between condition of device operating and not-operating. Within the tested ROI of images, no significant differences were observed among the SNR (*p* = 0.73) and the SFNR parameters (*p* = 0.67) of functional images (TABLE III).

### C. The Brain Response to Foot-Sole Pressure Simulator

**Fig. 6.**
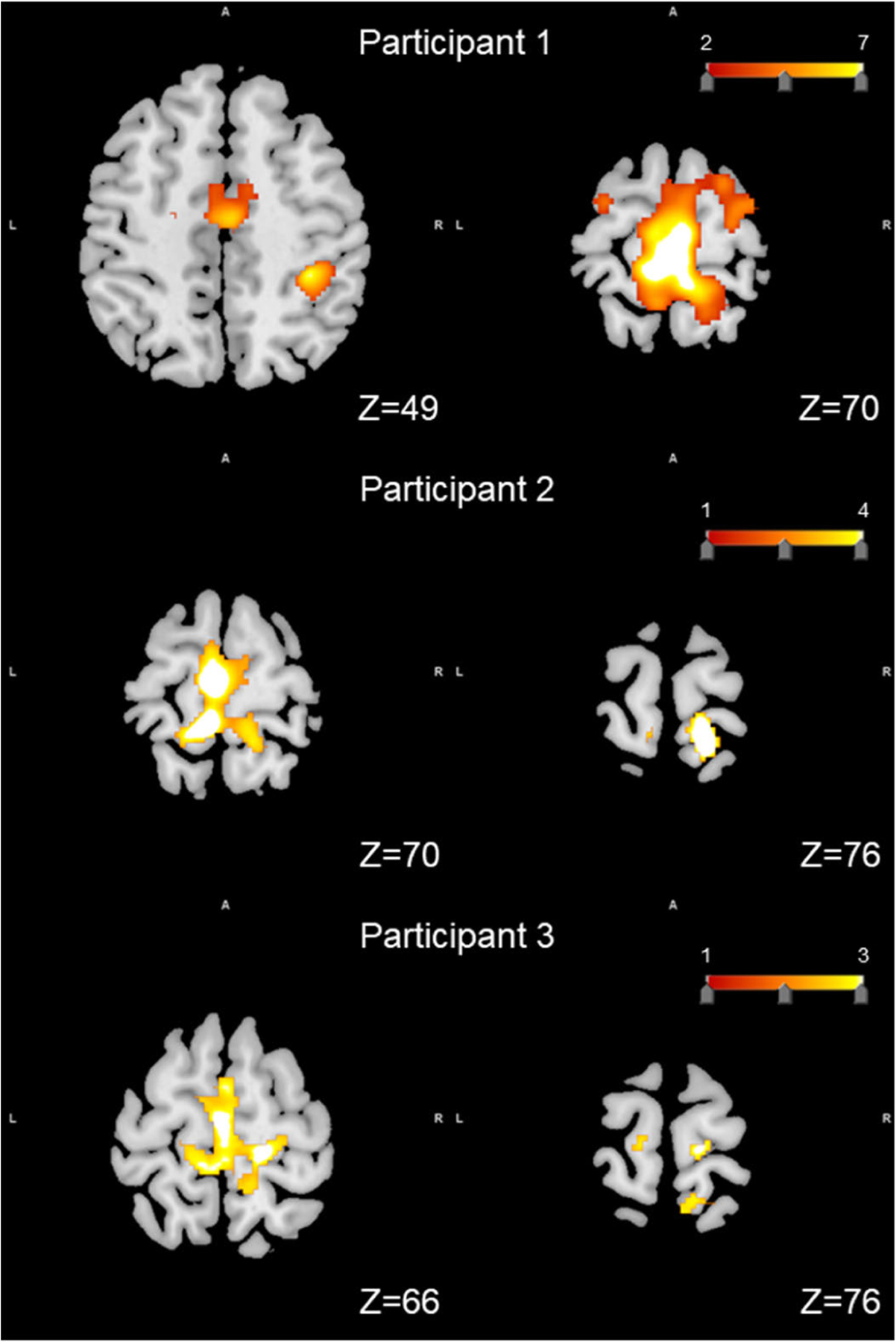
Activated regions of the brain overlaid on a standard T1 template in response to foot-sole pressure simulation in each participant. Across all three participants, the walking-related pressure stimuli was associated with significantly increased BOLD signal intensity within postcentral gyrus (Participant 1: Z=70; Participant 2: Z=76; Participant 3: Z=76), the supplementary motor area (SMA), precuneus gyrus, and paracentral lobule (Participant 1 and 2: Z=70; Participant 3: Z=66) (uncorrected p*<*0.01). Uniquely, in Participant 1, the activation in the superior parietal lobule (Z=70), superior frontal gyrus (Z=70), cingulate cortex (Z=49), and supramarginal gyrus (Z=49) was also observed (*p <*0.0005).

Fig. 6 shows the fMRI activation maps related to the response to the foot-sole stimulation as delivered by this stimulator in each participant. Across all participants, the intensity of the BOLD signal of postcentral gyrus, the supplementary motor area (SMA), precuneus gyrus, and paracentral lobule was significantly greater compared to resting blocks (uncorrected *p <* 0.05) (Fig. 6). Uniquely, in Participant 1, the activation in the superior parietal lobule, superior frontal gyrus, cingulate cortex, and supramarginal gyrus was also observed (*p <* 0.005).

## V. Discussion

In this work, we developed a novel MRI-compatible foot-sole stimulation system. Study results demonstrate that this stimulator can accurately and reliably simulate and apply the pressure changes (primarily on heel and forefoot of soles) and bilateral step times as experienced when walking over the ground, yet while the individual is lying motionless during the MRI scan and without interfering with the imaging quality. Additionally, preliminary evidence suggests that such walking-related pressure stimuli activate multiple supraspinal regions. Taken together, this device can serve as a novel tool helping characterize the brain activation pertaining to the sensorimotor control of walking.

Unlike previous MRI-compatible stimulators that applied single-point or bilateral stimulation [24], [25], [26], the system we developed simulates the transition of pressures from heel to forefoot on both feet that closely replicate and are directly proportional to what an individual actually experiences when walking. Compared to previous devices using one D/A conversion modular for both valves [17], separate D/A conversion modular is used for each valve to receive the command from the control unit and covert the programed digital signal of pressure into analog signals to form the motion of each proportional valve (i.e., executive unit) without delay. Therefore, a more timely and precise simulation of the pressure changes across the foot sole to that during overground walking can be achieved. This is supported by the results of this study, that is, the Spearman’s correlation coefficient (rho) on relationship between 0.70 and 0.90 in previous study [17], which, however, is at least 0.94 in this study. Meanwhile, such bilateral stimulation did not affect the imaging quality of MRI as evidenced by similar SNR and SFNR between power-on and-off condition, and such SNR and SFNR are also similar to that are reported in previous studies [17], [18]. Additionally, most previous similar devices were designed to only stimulate one foot and were thus incapable of simulating important bipedal gait characteristics (e.g., step time) that are known to be affected by aging and age-related conditions [27], [28], [29], [30], [31]. This novel functionality further enables boarder scenarios of its future application, such as simulating the gait characteristics in individuals suffering from neurological conditions that affect gait regulation (e.g., Parkinson’s disease) [32], [33].

In the fMRI brain activation study, we obtain preliminary evidence that multiple brain regions are activated in response to the walking-related foot-sole stimulation as applied by this stimulator. These regions include the postcentral gyrus where the primary somatosensory cortex locates, the precuneus gyrus that is closely connected to sensorimotor anterior, visual posterior and cognitive central regions, the paracentral lobule for the motor and sensory innervations of the contralateral lower extremity, and the SMA [34], [35], [36], [37]. This is consistent with previous studies exploring the brain activation as induced by MRI-compatible sensory stimulation systems [8], [17], [18], [23]. For example, Gallasch et al., observed that using the MRI-compatible vibrotactile stimulation system can induce significant activation in SMA [23]. Interestingly, in one participant, we observed the activation in additional regions, including the superior parietal lobule pertaining to spatial orientation and cognitive functions (e.g., attention) critical for sensorimotor control of walking [28], [29]; the superior frontal gyrus, associated with self-awareness in coordination with the sensory perception() [30], [31]; the cingulate cortex, linked to motivation [42]; and the supramarginal gyrus, a component of the somatosensory association cortex responsible for interpreting tactile sensation and spatial perception of limbs location [43]. Taken together, our preliminary observations based upon a very small group of participants indicate that these regions may contribute to the supraspinal, somatosensory control of walking. Still, many factors may influence the supraspinal activation pertaining to the sensorimotor control of walking, such as the brain structural atrophy [44], [45]. Therefore, future studies with larger sample size and explicit characterization of those underlying contributors are highly demanded to examine and confirm the supraspinal activation in response to the walking-related foot-sole stimulation as induced by this device.

It should be noted that even within one person, the temporal characteristics of gait change frequently within one bout of walking. Therefore, it would be helpful in future studies to track or monitor the pressure over a longer course of time, together with the tracking of other important characteristics contributing to the sensorimotor control of walking (e.g., dietary, mood, etc.). This may then enable more appropriate estimation and replication of walking-related pressure changes by using novel machine learning techniques for the individual. To avoid the head motion during MRI scan, we used simulated pressure stimuli of lower magnitude (i.e., 30% of body weight) as compared to that of overground walking. Therefore, it is unclear if and/or how the magnitudes of the pressure may influence the supraspinal activation. Future study is thus needed to directly compare the effects of different magnitudes (without inducing significant head motion affecting the image quality) of pressure on the supraspinal activation. Additionally, this system applies the pressure changes and related motion/rotation of foot in one degree of freedom, which is still not exactly the same as that during overground walking (e.g., three directional ration of ankle). Therefore, to more comprehensively understand the supraspinal sensorimotor control of walking, advanced functionalities of this MRI-compatible system (e.g., higher freedom of motion and rotation without interfering with the imaging quality) are needed in its future development. Nevertheless, this novel MRI-compatible foot-sole stimulation system enables the characterization of the supraspinal sensorimotor control pertaining to walking in aging and neurological conditions (e.g., peripheral neuropathy) and can help the understanding of pathways through which the rehabilitative strategies targeting the supraspinal elements for the performance of walking.

## Figures and Tables

**TABLE I:** The Results of Spearman’S Correlation Analysis for Force Change in Each Participant

	Heel		Forefoot	
	Left	Right	Left	Right
Participant 1	rho=0.95***	rho=0.96***	rho=0.97***	rho=0.95***
Participant 2	rho=0.96***	rho=0.97***	rho=0.98***	rho=0.94***
Participant 3	rho=0.94***	rho=0.95***	rho=0.98***	rho=0.98***
Participant 4	rho=0.95***	rho=0.96***	rho=0.96***	rho=0.96***
Participant 5	rho=0.98***	rho=0.97***	rho=0.98***	rho=0.98***
Participant 6	rho=0.96***	rho=0.95***	rho=0.97***	rho=0.97***
Participant 7	rho=0.95***	rho=0.95***	rho=0.95***	rho=0.95***

***: P<0.0001

**TABLE II:** The Step Time (Mean ± SD) as Produced by This Stimulation System and That of Overground Walking in Each Participant

	Overground Walking Step Time *(ms)*	Simulated Step Time *(ms)*
Participant 1	76.3±6.5	74.0±5.9
Participant 2	76.9±6.6	75.7±6.3
Participant 3	49.4±3.2	49.7±4.8
Participant 4	70.1±5.4	70.0±5.4
Participant 5	59.3±5.1	58.8±5.2
Participant 6	54.6±2.6	56±2.8
Participant 7	55.7±3.9	55.0±3.8

**TABLE III:** The SNR and SFNR of Phantom Images in “on” and “off” Condition

Mean+SD	ON	OFF	p value
SNR	29.84±4.32	29.78±3.76	0.73
SFNR	645±48	640±52	0.67

SNR: signal-to-noise ratio; SNFR: signal-to-fluctuation noise ratio

## References

[1] Eils E, Behrens S, Mers O, Thorwesten L, Völker K, and Rosenbaum D, “Reduced plantar sensation causes a cautious walking pattern,” Gait Posture, vol. 20, no. 1, pp. 54–60, Aug. 2004. 15196521 10.1016/S0966-6362(03)00095-X

[2] Chen Y-S and Zhou S, “Soleus H-reflex and its relation to static postural control,” Gait Posture, vol. 33, no. 2, pp. 169–178, Feb. 2011. 21211976 10.1016/j.gaitpost.2010.12.008

[3] Favorov OV, Nilaweera WU, Miasnikov AA, and Beloozerova IN, “Activity of somatosensory-responsive neurons in high subdivisions of Si cortex during locomotion,” J. Neurosci, vol. 35, no. 20, pp. 7763–7776, May 2015. 25995465 10.1523/JNEUROSCI.3545-14.2015 PMC4438126

[4] Sangani S, Lamontagne A, and Fung J, “Cortical mechanisms underlying sensorimotor enhancement promoted by walking with haptic inputs in a virtual environment,” Prog. Brain Res, vol. 218, no. 2, pp. 313–330, 2015. 25890144 10.1016/bs.pbr.2014.12.003

[5] Gwin JT, Gramann K, Makeig S, and Ferris DP, “Electrocortical activity is coupled to gait cycle phase during treadmill walking,” NeuroImage, vol. 54, no. 2, pp. 1289–1296, Jan. 2011. 20832484 10.1016/j.neuroimage.2010.08.066

[6] Clark DJ, Christou EA, Ring SA, Williamson JB, and Doty L, “Enhanced somatosensory feedback reduces prefrontal cortical activity during walking in older adults,” Journals Gerontol. Ser. A, Biol. Sci. Med. Sci, vol. 69, no. 11, pp. 1422–1428, Nov. 2014. 10.1093/gerona/glu125 PMC4229993 25112494

[7] Knikou M, “Neural control of locomotion and training-induced plasticity after spinal and cerebral lesions,” Clin. Neurophysiol, vol. 121, no. 10, pp. 1655–1668, Oct. 2010. 20427232 10.1016/j.clinph.2010.01.039

[8] Hong Y, Bao D, Manor B, and Zhou J, “Characterizing the supraspinal sensorimotor control of walking using MRI-compatible system: A systematic review,” J. NeuroEng. Rehabil, vol. 21, no. 1, pp. 1–20, Mar. 2024. 38443983 10.1186/s12984-024-01323-y PMC10913571

[9] Holtzer R, Mahoney JR, Izzetoglu M, Izzetoglu K, Onaral B, and Verghese J, “fNIRS study of walking and walking while talking in young and old individuals,” J. Gerontol. Ser. A, Biomed. Sci. Med. Sci, vol. 66, no. 8, pp. 879–887, Aug. 2011. 10.1093/gerona/glr068 PMC3148759 21593013

[10] Dobkin BH, Firestine A, West M, Saremi K, and Woods R, “Ankle dorsiflexion as an fMRI paradigm to assay motor control for walking during rehabilitation,” NeuroImage, vol. 23, no. 1, pp. 370–381, Sep. 2004. 15325385 10.1016/j.neuroimage.2004.06.008 PMC4164211

[11] Gwin JT, Gramann K, Makeig S, and Ferris DP, “Removal of movement artifact from high-density EEG recorded during walking and running,” J. Neurophysiol, vol. 103, no. 6, pp. 3526–3534, Jun. 2010. 20410364 10.1152/jn.00105.2010 PMC3774587

[12] Kwong KK “Dynamic magnetic resonance imaging of human brain activity during primary sensory stimulation,” Proc. Nat. Acad. Sci. USA, vol. 89, no. 12, pp. 5675–5679, Jun. 1992. 1608978 10.1073/pnas.89.12.5675 PMC49355

[13] Glover GH, “Overview of functional magnetic resonance imaging,” Neurosurg. Clin. North Amer, vol. 22, no. 2, pp. 133–139, 2011. 10.1016/j.nec.2010.11.001 PMC3073717 21435566

[14] Yu N, Hollnagel C, Blickenstorfer A, Kollias SS, and Riener R, “Comparison of MRI-compatible mechatronic systems with hydrodynamic and pneumatic actuation,” IEEE/ASME Trans. Mechatronics, vol. 13, no. 3, pp. 268–277, Jun. 2008.

[15] Ueda J “MRI—Compatible fluid-powered medical devices,” Mech. Eng, vol. 135, no. 6, pp. 13–16, Jun. 2013.

[16] Tsekos NV, Khanicheh A, Christoforou E, and Mavroidis C, “Magnetic resonance-compatible robotic and mechatronics systems for image-guided interventions and rehabilitation: A review study,” Annu. Rev. Biomed. Eng, vol. 9, no. 1, pp. 351–387, Aug. 2007. 17439358 10.1146/annurev.bioeng.9.121806.160642

[17] Zhang T “An MRI-compatible foot-sole stimulation system enabling characterization of the brain response to walking-related tactile stimuli,” Frontiers Neurosci, vol. 13, p. 1075, Oct. 2019. 10.3389/fnins.2019.01075 PMC6811610 31680815

[18] Hao Y “Novel MRI-compatible tactile stimulator for cortical mapping of foot sole pressure stimuli with fMRI,” Magn. Reson. Med, vol. 69, no. 4, pp. 1194–1199, Apr. 2013. 22678849 10.1002/mrm.24330 PMC3704153

[19] Friedman L and Glover GH, “Report on a multicenter fMRI quality assurance protocol,” J. Magn. Reson. Imag, vol. 23, no. 6, pp. 827–839, Jun. 2006. 10.1002/jmri.20583 16649196

[20] Reber PJ, Wong EC, Buxton RB, and Frank LR, “Correction of off resonance-related distortion in echo-planar imaging using EPI-based field maps,” Magn. Reson. Med, vol. 39, no. 2, pp. 328–330, Feb. 1998. 9469719 10.1002/mrm.1910390223

[21] Windischberger C, Robinson S, Rauscher A, Barth M, and Moser E, “Robust field map generation using a triple-echo acquisition,” J. Magn. Reson. Imag. Jmri, vol. 20, no. 4, pp. 730–734, Sep. 2004. 10.1002/jmri.20158 15390143

[22] Menz HB, Latt MD, Tiedemann A, Mun San Kwan M, and Lord SR, “Reliability of the GAITRite^®^ walkway system for the quantification of temporo-spatial parameters of gait in young and older people,” Gait Posture, vol. 20, no. 1, pp. 20–25, Aug. 2004. 15196515 10.1016/S0966-6362(03)00068-7

[23] Altman DG and Bland JM, “Measurement in medicine: The analysis of method comparison studies,” J. Roy. Statist. Soc. D, Statistician, vol. 32, no. 3, pp. 307–317, Sep. 1983.

[24] Gallasch E “Contact force- and amplitude-controllable vibrating probe for somatosensory mapping of plantar afferences with fMRI,” J. Magn. Reson. Imag, vol. 24, no. 5, pp. 1177–1182, Nov. 2006. 10.1002/jmri.20742 17031838

[25] Hollnagel E, “The four cornerstones of resilience engineering,” in Resilience Engineering Perspectives. USA: CRC Press, 2009, pp. 139–156. Accessed: Apr. 4. 2024.

[26] Hartwig V, Carbonaro N, Tognetti A, and Vanello N, “Systematic review of fMRI compatible devices: Design and testing criteria,” Ann. Biomed. Eng, vol. 45, no. 8, pp. 1819–1835, Aug. 2017. 28550499 10.1007/s10439-017-1853-1

[27] Olney SJ and Richards C, “Hemiparetic gait following stroke. Part I: Characteristics,” Gait. Posture, vol. 4, no. 2, pp. 136–148, Apr. 1996.

[28] Zijlstra W, Rutgers AWF, and Van Weerden TW, “Voluntary and involuntary adaptation of gait in Parkinson’s disease,” Gait Posture, vol. 7, no. 1, pp. 53–63, Jan. 1998. 10200376 10.1016/s0966-6362(97)00037-4

[29] Salarian A “Gait assessment in Parkinson’s disease: Toward an ambulatory system for long-term monitoring,” IEEE Trans. Biomed. Eng, vol. 51, no. 8, pp. 1434–1443, Aug. 2004. 15311830 10.1109/TBME.2004.827933

[30] Farashi S, “Analysis of the stance phase of the gait cycle in Parkinson’s disease and its potency for Parkinson’s disease discrimination,” J. Biomechanics, vol. 129, Dec. 2021, Art. no. 110818. 10.1016/j.jbiomech.2021.110818 34736084

[31] Wuehr M “Sensory loss and walking speed related factors for gait alterations in patients with peripheral neuropathy,” Gait Posture, vol. 39, no. 3, pp. 852–858, Mar. 2014. 24342450 10.1016/j.gaitpost.2013.11.013

[32] Balaban B and Tok F, “Gait disturbances in patients with stroke,” PM R, vol. 6, no. 7, pp. 635–642, Jul. 2014. 24451335 10.1016/j.pmrj.2013.12.017

[33] Von Schroeder HP, Coutts RD, Lyden PD, Billings E, and Nickel VL, “Gait parameters following stroke: A practical assessment,” J. Rehabil. Res. Develop, vol. 32, p. 25, Feb. 1995. 7760264

[34] Bittar RG, Olivier A, Sadikot AF, Andermann F, Pike GB, and Reutens DC, “Presurgical motor and somatosensory cortex mapping with functional magnetic resonance imaging and positron emission tomography,” J. Neurosurg, vol. 91, no. 6, pp. 915–921, Dec. 1999. 10584835 10.3171/jns.1999.91.6.0915

[35] Goldberg G, “Supplementary motor area structure and function: Review and hypotheses,” Behav. Brain Sci, vol. 8, no. 4, pp. 567–588, Dec. 1985.

[36] Cavanna AE and Trimble MR, “The precuneus: A review of its functional anatomy and behavioural correlates,” Brain, vol. 129, no. 3, pp. 564–583, Mar. 2006. 16399806 10.1093/brain/awl004

[37] Patra A, Kaur H, Chaudhary P, Asghar A, and Singal A, “Morphology and morphometry of human paracentral lobule: An anatomical study with its application in neurosurgery,” Asian J. Neurosurg, vol. 16, no. 2, pp. 349–354, Jun. 2021. 34268163 10.4103/ajns.AJNS_505_20 PMC8244697

[38] Caminiti R, Ferraina S, and Johnson PB, “The sources of visual information to the primate frontal lobe: A novel role for the superior parietal lobule,” Cerebral Cortex, vol. 6, no. 3, pp. 319–328, 1996. 8670660 10.1093/cercor/6.3.319

[39] Felician O “The role of human left superior parietal lobule in body part localization,” Ann. Neurol, vol. 55, no. 5, pp. 749–751, May 2004. 15122719 10.1002/ana.20109

[40] Boisgueheneuc FD “Functions of the left superior frontal gyrus in humans: A lesion study,” Brain, vol. 129, no. 12, pp. 3315–3328, Jun. 2006. 16984899 10.1093/brain/awl244

[41] Hu S, Ide JS, Zhang S, and Li C-S-R, “The right superior frontal gyrus and individual variation in proactive control of impulsive response,” J. Neurosci, vol. 36, no. 50, pp. 12688–12696, Dec. 2016. 27974616 10.1523/JNEUROSCI.1175-16.2016 PMC5157110

[42] Devinsky O, Morrell MJ, and Vogt BA, “Contributions of anterior cingulate cortex to behaviour,” Brain, vol. 118, no. 1, pp. 279–306, Feb. 1995. 7895011 10.1093/brain/118.1.279

[43] Riva F, Lenger M, Kronbichler M, Lamm C, and Silani G, “The role of right supra-marginal gyrus and secondary somatosensory cortex in age-related differences in human emotional egocentricity,” Neurobiol. Aging, vol. 112, pp. 102–110, Apr. 2022. 35104721 10.1016/j.neurobiolaging.2022.01.002

[44] Kalpouzos G, Persson J, and Nyberg L, “Local brain atrophy accounts for functional activity differences in normal aging,” Neurobiol. Aging, vol. 33, no. 3, p. 623, Mar. 2012. 10.1016/j.neurobiolaging.2011.02.021 21524432

[45] Johnson SC “The relationship between fMRI activation and cerebral atrophy: Comparison of normal aging and Alzheimer disease,” NeuroImage, vol. 11, no. 3, pp. 179–187, Mar. 2000. 10694460 10.1006/nimg.1999.0530

